# Factors Affecting the Frequency of Maxillofacial Injuries in Jahrom, Iran 

**DOI:** 10.30476/dentjods.2025.105054.2566

**Published:** 2025-12-01

**Authors:** Amirhossein Samiee Dehpagaee, Esmail Rayatdoost

**Affiliations:** 1 Research Center for Noncommunicable Diseases, Jahrom University of Medical Sciences, Jahrom, Iran.

**Keywords:** Wounds and Injuries, Trauma Centers, Prevalence, Epidemiologic Factors, Iran

## Abstract

**Background:**

Maxillofacial trauma constitutes a major public health concern due to its potential for severe complications and substantial impact on quality of life.

**Purpose:**

This study aimed to elucidate the factors influencing the occurrence of maxillofacial trauma in patients presenting with traumatic injuries at Peymaniyeh Hospital in Jahrom, Iran.

**Materials and Method:**

This cross-sectional study examined trauma patients at Peymaniyeh Hospital in Jahrom, Iran, from April 2022 to March 2023. A census approach included all trauma patients whose data were recorded in the National Trauma Registry of Iran. Data were collected using a tailored checklist that captured demographic and contextual variables along with injury mechanisms. Statistical analyses were performed using SPSS version 22.

**Results:**

Of 924 trauma patients, 218 were diagnosed with maxillofacial trauma and 706 with non-maxillofacial trauma. The majority of the trauma patients were male (76.4%), and the distribution of age groups was not statistically different between the two types of injuries (*p*= 0.571). Univariate analysis revealed that substance (*p*= 0.007) and alcohol use (*p*< 0.001), road traffic accidents (RTAs) (*p*= 0.001), and representation of motorcyclists (*p*= 0.001) were significantly more prevalent in maxillofacial injury compared to non-maxillofacial injury category. Logistic regression analysis revealed that substance use (OR= 2.04, 95% CI 1.03-3.99, *p*= 0.040), alcohol consumption (OR= 2.89, 95% CI 1.37-6.09, *p*= 0.005), and experiencing RTAs (OR= 12.80, 95% CI 6.07-26.98, *p*< 0.001) and falling (OR= 3.69, 95% CI 1.68-8.11, *p*= 0.001) were significantly associated with the occurrence of maxillofacial injuries.

**Conclusion:**

This study underscores the prominence of RTAs, particularly those involving motorcyclists, as a primary cause of maxillofacial injuries. The significant association between alcohol and substance use and the elevated risk of these injuries is evident. These findings highlight the need for targeted prevention strategies to promote safe driving practices, and implement public health policies aimed at mitigating alcohol and substance use disorder to reduce the incidence of such injuries.

## Introduction

Maxillofacial trauma represents a significant global health issue, characterized by considerable variations in prevalence across different regions. According to a 2019 report from the World Health Organization (WHO), injuries result in over 4.3 million fatalities annually, averaging approximately 11,780 deaths daily [ 1 ]. Notably, low- and middle-income countries account for 89% of these fatalities [ 2 ]. The incidence of maxillofacial fractures is disproportionately higher among males, constituting 81.04% of cases. The most affected age group is between 21-30 years, comprising 43.23% of the total number of maxillofacial fractures [ 3 ].

Maxillofacial injuries can arise from a diverse array of mechanisms, including road traffic accidents (RTAs), interpersonal violence, falls, and sports-related incidents [ 4 ]. The severity and distribution of maxillofacial injuries are contingent upon the anatomical location of the trauma, the intensity of the impact, and the angle at which the force is applied to the face [ 5 ]. In instances of maxillofacial injuries, 64% of cases involved isolated fractures of the mandible, whereas 19% of cases involved isolated midface fractures [ 6 ]. In Iran, RTAs constitute the predominant cause of maxillofacial trauma (68.9%), followed by falls (12.62%), accounting for a significant proportion of cases [ 3 ]. 

Demographic factors significantly influence the occurrence of maxillofacial trauma. Studies indicate that males, particularly those age group 21-30 years, are disproportionately affected [ 3 , 7 - 9 ]. Socioeconomic status also emerges as a critical risk indicator for maxillofacial injuries. Individuals from lower socioeconomic status backgrounds often experience higher rates of trauma due to limited access to healthcare, increased engagement in high-risk activities, and inadequate safety measures [ 10 ]. Studies further elucidate the prevalence of alcohol consumption and substance use among patients with maxillofacial injuries, highlighting how intoxication can impair judgment and elevate the risk of accidents [ 11 - 12 ]. Moreover, risk-taking behaviors, such as neglecting to use protective gear and participating in dangerous activities, markedly increase the likelihood of maxillofacial injuries, particularly among adolescents and young adults [ 13 - 14 ]. Additionally, the trauma sustained may be associated with brain injuries and other critical areas, such as the eyes [ 15 ]. 

The objective of this study was to investigate the factors influencing the occurrence of maxillofacial trauma in trauma patients referred to the Peymaniyeh Hospital in Jahrom. By analyzing patient demographics, mechanisms of injury, and associated factors, this study aimed to provide a comprehensive overview of the current state of maxillofacial trauma in this region. The findings aim to not only contribute to the existing body of literature but also serve as a foundation for future studies endeavoring to improve patient outcomes and reduce the incidence of these traumatic injuries.

## Materials and Method

This cross-sectional study focused on all trauma patients who were transferred or referred to the emergency room of Peymaniyeh Hospital in Jahrom, a city situated in the central district of Jahrom County, Fars Province, Iran. Utilizing a census approach, we included trauma patients whose details were recorded in the National Trauma Registry of Iran. The study encompassed individuals who were hospitalized for at least 24 hours, those who died in the emergency department within 24 hours of hospitalization, and patients transferred from the special care department of another hospital to the intensive care unit of Peymaniyeh Hospital. Conducted based on secondary data analysis from the National Trauma Registry of Iran, the study spanned a 12-month period from April 2022 to March 2023.

Data were gathered using a checklist specifically created to align with the research objectives. This form extracts key variables, including demographic information such as age, sex, marital status, and education level, as well as contextual variables such as drug use, alcohol consumption, and sedative use. Additionally, the mechanism of injury was classified into categories such as RTAs, falls from a height, and other specified causes.

The mechanisms of injury were delineated as follows: RTAs, which involve collisions between vehicles; falls from a height, defined by the WHO [ 16 ] as any accident where an individual falls to a lower level.

Ethical considerations for this study encompassed ensuring patient confidentiality by anonymizing data, obtaining the necessary approval from relevant ethics committees, and ensuring informed consent where applicable. Additionally, this study aimed to minimize any potential harm by using secondary data and focusing on the analysis of existing records, rather than direct patient interaction. The research conducted by the Jahrom University of Medical Sciences was registered under the code IR.JUMS.REC.1402.109.

### Statistical analysis

Data were analyzed using IBM SPSS for Windows version 22 software (Armonk, NY, IBM Corp.). Descriptive statistics, including frequency, percentage, and mean and standard deviation (as mean ± SD) were computed to summarize the characteristics of the sample. Additionally, Pearson’s chi-square test was used to assess associations between categorical variables and type of injury (univariate analysis). Additionally, multiple logistic regression analysis was employed to examine the association between the potential predictor variables and the occurrence of jaw and facial trauma. The potential predictors were defined as the variables yielded a *p* value (P) less than 0.200 in univariate analyses. Odds ratios (OR) and 95% confidence intervals (CI) were reported from logistic regression method. The significance level was set at α= 0.05 for all analyses.

## Results

The study included 924 trauma patients, of which 218 experienced maxillofacial trauma, and 706 did not. The majority of the trauma patients were male (76.40%).

### Sociodemographic characteristics

The mean age of participants in the maxillofacial group (38.15 years) was not significantly different from that in the non-maxillofacial group (40.42 years) (*p*= 0.21). Moreover, the occurrence of maxillofacial trauma was not statistically different between age groups (*p*= 0.571). In terms of sex distribution, a higher percentage of males were observed in both groups: 80.7% in the maxillofacial group compared to 75.1% in the non-maxillofacial group, although sex ratio was not statistically different between the two types of traumas (*p*= 0.085). Marital status was not statistically associated with types of traumas (*p*= 0.055). The majority of participants in both groups were married, with 52.3% in the maxillofacial group and 59.6% in the non-maxillofacial group. The two groups were not statistically different in terms of educational status (*p*= 0.831). The most prevalent educational status was diploma and under diploma degrees in both groups. Table 1 summarizes the sociodemographic characteristics of all participants, providing a comprehensive overview of key variables such as age, gender, marital status and educational level.

**Table 1:** Sociodemographic characteristics of all participants

Variable		Maxillofacial trauma (N= 218)	Non-maxillofacial trauma (N=706)	*p* Value
Age (Mean ±SD)		38.15 ±22.49	40.42 ±23.66	0.210
Age group	<20	58 (26.6%)	182 (25.8%)	0.571
	20-29	35 (16.1%)	86 (12.2%)	
	30-39	36 (16.5%)	114 (16.1%)	
	40-49	24 (11.0%)	87 (12.3%)	
	≥50	65 (29.8%)	237 (33.6%)	
Sex	Male	176 (80.7%)	530 (75.1%)	0.085
	Female	42 (19.3%)	176 (24.9%)	
Marital status	Married	114 (52.3%)	421 (59.6%)	0.055
	Single	104 (47.4%)	285 (40.4%)	
Educational status	Diploma and lower	155 (71.1%)	492 (69.7%)	0.831
	Illiterate	53 (24.3%)	185 (26.2%)	
	Higher than diploma	10 (4.6%)	29 (4.1%)	

N: Number; SD: Standard deviation

### Substance and alcohol use

Table 2 shows the association between substance and al-cohol use with type of trauma. Substance use was higher among patients with maxillofacial trauma (7.8%) compared to those with non-maxillofacial trauma (3.5%) (*p*= 0.007). Similarly, alcohol use was more prevalent in maxillofacial trauma patients (8.8%) than those with non-maxillofacial trauma (2.8%) (*p*< 0.001). However, the frequency of sedative usage was trivial and similar in both groups, with only 0.9% of maxillofacial trauma patients and 1.3% of non-maxillofacial trauma patients using them (*p*= 0.676).

**Table 2:** Substance, Alcohol, and Sedative use among maxillofacial and non-maxillofacial trauma patients

Variable		Maxillofacial trauma (N=218)	Non-maxillofacial trauma (N=706)	*p* Value
Substance use	Yes	17 (7.8%)	25 (3.5%)	0.007
	No	201 (92.2%)	681 (96.5%)	
Alcohol use	Yes	19 (8.8%)	20 (2.8%)	<0.001
	No	199 (91.2%)	686 (97.2%)	
Sedative use	Yes	2 (0.9%)	9 (1.3%)	0.676
	No	216 (99.1%)	697 (98.7%)	

N: Number

### Trauma-related factors

Table 3 shows the association between trauma-related factors as well as safety measure use in RTAs with type of trauma. The mechanisms of injury differed significantly between the two groups (*p*= 0.001). The difference was more remarkable in the context of RTAs; a majority of maxillofacial trauma cases (71.6%) were due to RTAs, compared to only 33.7% in the non-maxillofacial group. The situation of the person in trauma was statistically associated with type of trauma (*p*= 0.001).

**Table 3:** Trauma-related factors and safety measure utilization in RTAs among maxillofacial and non-maxillofacial trauma patients

Variable			Maxillofacial trauma	Non-maxillofacial trauma	*p* Value
Mechanism of injury		RTAs	156 (71.6%)	238 (33.7%)	0.001
		Non-stabbing blunt trauma	7 (3.2%)	50 (7.1%)	
		Falls from a height	47 (21.6%)	263 (37.3%)	
		Stabbings or cuts	3 (1.4%)	136 (19.3%)	
		Animal attack	1 (0.5%)	4 (0.6%)	
		Suffocation	0 (0%)	2 (0.3%)	
		Electric injury	0 (0%)	1 (0.1%)	
		Gunshot	3 (1.4%)	1 (0.1%)	
		Facing the blast wave	1 (0.5%)	0 (0%)	
		Unknown	0 (0%)	11 (1.6%)	
		Total	218 (100%)	706 (100%)	
Situation of the person in trauma a		Pedestrian	15 (9.6%)	35 (14.7%)	0.001
		Bicyclist	1 (0.6%)	2 (0.8%)	
		Motorcyclist	121 (77.6%)	139 (58.4%)	
		Car occupant	18 (11.5%)	60 (25.2%)	
		Heavy vehicle occupant	1 (0.6%)	2 (0.8%)	
		Total	156 (100%)	238 (100%)	
Traumatic person role ^a^		Passenger	104 (73.8%)	160 (78.8%)	0.274
		Driver	37 (26.2%)	43 (21.2%)	
		Total	141 (100%)	203 (100%)	
Accident type ^a^		Collision	98 (62.8%)	155 (65.1%)	0.641
		Vehicle overturning	58 (37.2%)	83 (34.9%)	
		Total	156 (100%)	238 (100%)	
Opposite object ^a^		Bicycle	0 (0%)	1 (0.7%)	0.664
		Motorcycle	15 (15.5%)	25 (16.3%)	
		Passenger car	71 (73.2%)	117 (76.5%)	
		Heavy vehicle	2 (2.1%)	2 (1.3%)	
		Fixed object	9 (9.3%)	8 (5.2%)	
		Total	97 (100%)	153 (100%)	
Intentional traumatic injuries		Yes	8 (3.7%)	45 (6.4%)	0.135
		No	210 (96.3%)	661 (93.6%)	
		Total	218 (100%)	706 (100%)	
Cause of trauma (in intentional traumatic injuries)		Violence/conflict	8 (100%)	38 (84.4%)	0.231
		Suicide/self-harm	0 (0%)	7 (15.6%)	
		Total	8 (100%)	45 (100%)	
Safety measure use ^a^	Seat belt b	Yes	11 (57.9%)	6 (9.7%)	0.000
		No	8 (42.1%)	56 (90.3%)	
		Total	19 (100%)	62 (100%)	
	Child safety seat ^b^	Yes	0 (0%)	0 (0%)	NA
		No	19 (100%)	62 (100%)	
		Total	19 (100%)	62 (100%)	
	Airbag ^b^	Yes	0 (0%)	0 (0%)	NA
		No	19 (100%)	62 (100%)	
		Total	19 (100%)	62 (100%)	
	Helmet ^c^	Yes	1 (0.8%)	2 (1.4%)	0.999
		No	121 (99.2%)	139 (98.6%)	
		Total	122 (100%)	141 (100%)	
Season of trauma		Spring	49 (22.5%)	168 (23.8%)	0.776
		Summer	72 (33%)	252 (35.7%)	
		Autumn	63 (28.9%)	187 (26.5%)	
		Winter	34 (15.6%)	99 (14%)	
		Total	218 (100%)	706 (100%)	

RTAs: Road traffic accidents; ^a^ Applicable only to RTA victims; ^b^ Applicable only to car/heavy vehicle occupants; ^c^ Applicable only to bicyclists and motorcyclists

In this context, notably, the prominent difference belonged to motorcyclist category followed by car occupant category; a higher percentage of individuals involved in maxillofacial trauma were motorcyclists (77.6%) compared to those in non-maxillofacial trauma (58.4%). Conversely, a lower proportion of car occupants were involved in maxillofacial trauma (11.5%) than in non-maxillofacial trauma (25.2%). However, the role of the traumatic person (*p*= 0.274), accident types (*p*= 0.641), occurrence of intentional traumatic injuries (*p*= 0.135) and its causes of trauma (*p*= 0.231), and season of trauma (*p*= 0.776) showed no significant difference between the two groups.

### Safety measures in RTAs

In terms of safety measures used in RTAs, the use of seat belts was reported by 57.9% of maxillofacial trauma victims (11 out of 19) compared to 9.7% of non-maxillofacial trauma cases (6 out of 62) (*p*= 0.000). However, helmet usage was similarly low across both groups (0.8% for maxillofacial vs. 1.4% for non-maxillofacial) (*p*= 0.999). The statistical analysis of other safety measures, such as child safety seats and airbags, was not applicable because none of the participants reported using them.

### Logistic regression analysis

Table 4 displays the findings from a logistic regression analysis using injury type as dependent variable and potential effective factors as predictors. The potential predictors were the variables yielded a p value less than 0.200 in univariate analyses reported in Tables 1-3. In this context, variables that were defined exclusively for patients with any RTA experience (such as seat belt usage, traumatic person role, accident type, etc.) did not include in this strategy. Substance (OR= 2.04, 95% CI: 1.03-3.99, *p*= 0.040) and alcohol use (OR= 2.89, 95% CI: 1.37-6.09, *p*= 0.005) were significantly associated with higher occurrence of maxillofacial injuries. Patients who engaged in substance use were 2.04 times more likely to have maxillofacial injuries compared to those who did not it. Similarly, the odds of experiencing maxillofacial injuries for those consuming alcohol was 2.89 times greater than that of patients who did not consume alcohol. Additionally, the maxillofacial injuries were more prevalent among those who either experienced RTAs (OR= 12.80, 95% CI: 6.07-26.98, *p*< 0.001) or falling (OR= 3.69, 95% CI: 1.68-8.11, *p*= 0.001) compared to those with other mechanism of injuries. In contrast, other factors such as sex, marital status, and intentional traumatic injuries did not exhibit significant association with maxillofacial injuries. 

**Table 4:** Logistic regression of selected variables for prediction of maxillofacial injuries

Variable		OR	95% CI	*p* Value
Sex	Female	1	-	-
	Male	0.91	0.58-1.42	0.671
Marital status	Single	1	-	-
	Married	0.90	0.64-1.28	0.562
Substance use	No	1	-	-
	Yes	2.04	1.03-3.99	0.040
Alcohol use	No	1	-	-
	Yes	2.89	1.37-6.09	0.005
Intentional traumatic injuries	No	1	-	-
	Yes	2.64	0.91-7.70	0.075
Mechanism of injury ^a^	Other	1	-	-
	RTAs	12.80	6.07-26.98	<0.001
	Fall	3.69	1.68-8.11	0.001

OR: Odds ratio; CI: Confidence interval; The reference category for each predictor, indicated by OR=1; ^a^ Due to the small sample size in certain mechanism of injury classes, we categorized them into three groups

## Discussion

This study investigated the determinants of maxillofacial trauma, providing an in-depth analysis of the differences between maxillofacial and non-maxillofacial trauma. Our findings indicate that RTAs are the primary cause of maxillofacial trauma, which is consistent with studies conducted in various countries, including Italy [ 17 ], China [ 18 ], and India [ 19 ]. Conversely, falls are the leading cause of non-maxillofacial trauma, followed by RTAs. These results highlight the distinct causality of maxillofacial versus non-maxillofacial injuries and emphasize the significant impact of RTAs in Jahrom. This observation corroborates previous research conducted at the Jahrom Trauma Center, which also identified RTAs and falls as the most prevalent mechanisms of trauma [ 20 ]. Additionally, our investigation reveals that the incidence of RTAs in Jahrom County mirrors national trends, which is higher than the global average [ 21 ]. Moreover, both stabbing and non-stabbing blunt trauma injuries, which were more common in the non-maxillofacial group, were less likely to cause maxillofacial trauma.

Our study demonstrated that the mean age of participants and the occurrence of maxillofacial trauma were not significantly different between age groups. Although a higher percentage of males were observed in both trauma groups, the difference between the two groups was not statistically significant. These findings suggest that gender and age may not be primary determinants of maxillofacial trauma in our sample population. However, the higher illiteracy rate observed in the maxillofacial group, although not statistically significant, aligns with the findings of Esses et al. [ 22 ], who reported a high rate of illiteracy among trauma patients. This indicates that low educational levels may be a contributing factor to trauma susceptibility.

This study elucidated that trauma circumstances varied significantly based on the situation of the person involved in RTAs. Specifically, pedestrians predominantly sustain non-maxillofacial injuries, while car occupants also exhibited a higher likelihood of experiencing non-maxillofacial trauma. Notably, for individuals utilizing bicycles or heavy vehicles, the circumstances did not show a difference. 

Consistent with previous studies, age and sex are key factors influencing the occurrence and type of road traffic injuries [ 23 - 24 ]. In our study, over 80% of maxillofacial injuries and 75% of non-maxillofacial injuries occurred in males, reflecting findings from other researches [ 25 - 26 ]. Men generally spend more hours driving and engage in riskier driving behaviors than women [ 26 ], which may explain the higher rate of driving-related injuries among men. Additionally, in our study, most maxillofacial and non-maxillofacial injuries were caused by motorcycles. Due to societal norms in Iran, although there is no legal prohibition against women obtaining a motorcycle license, they are rarely permitted to drive [ 27 ]. This likely contributes to the lower incidence of these injuries among women.

While alcohol and substance use are established risk factors for road traffic injuries, their impact on maxillofacial trauma remains less understood. Our comparison revealed a strong correlation between alcohol and substance use and the occurrence maxillofacial trauma. Lee et al. [ 28 ] revealed that 18% of patients with maxillofacial injuries had alcohol involvement, while other studies in the U.S. [ 29 ], New Zealand [ 30 ], and Australia [ 31 ] reported varying rates of alcohol involvement in maxillofacial trauma cases. Additionally, Othman et al. [ 32 ] found that a positive urine drug screen was associated with facial fractures compared to a negative screen. There is also a link between violent behavior and substance use, which can lead to violence-induced trauma [ 33 ]. Furthermore, substance use can lead to dangerous driving, explaining the relationship between substance use and RTAs [ 34 ].

Comprehensive data collection through a census approach ensured a broad representation of maxillofacial trauma cases, enhancing the findings' reliability. The application statistical rigor, including logistic regression analyses, provided a nuanced understanding of the associations between variables and the occurrence of maxillofacial trauma. By focusing on Jahrom County, Iran, this study provides valuable insights into local public health concerns and informs targeted interventions. Additionally, it highlights important demographic factors, such as gender and educational level, contributing to the understanding of the social determinants of health.

However, this study had some limitations. The cross-sectional design restricts the ability to draw causal inferences from the observed relationships. Additionally, reliance on secondary data may have introduced biases related to data accuracy and completeness, particularly concerning substance use and other sensitive variables. There is also a lack of follow-up data on the long-term outcomes of maxillofacial trauma, which could provide further context for recovery and quality of life. Finally, the findings may not be applicable to other regions or populations outside of Jahrom County, limiting the external validity of the results.

## Conclusion

In conclusion, this study effectively identified and analyzed the factors influencing the occurrence of maxillofacial trauma among patients referred to the Peymaniyeh Hospital in Jahrom, Iran. The principal findings suggest that RTAs are the predominant cause of such injuries, especially those involving motorcyclists, followed by falls. Furthermore, the study established a significant correlation between alcohol and substance use and the occurrence of maxillofacial injuries, underscoring the necessity of addressing these risk factors in preventive strategies. Despite certain limitations, this research provides critical insights that can inform public health policies and preventive measures aimed at mitigating the incidence of maxillofacial trauma in Iran. Future studies would benefit from employing longitudinal designs and expanding the geographical scope to enhance our understanding of this public health issue.
